# The Treatment of Syphilis

**Published:** 1904-07-23

**Authors:** 


### HOSPITAL CLINICS.

### THE TREATMENT OF SYPHILIS.

LEWIS WICKHAM [1] advocates mercurial injections in the treatment of syphilis. He claims that (1) this method relieves the stomach and intestines, and thus does not interfere with the digestive functions, making it possible to maintain a high standard of health. (2) The stomach is in a condition to receive potassium iodide when the time comes for its administration. (3) There is more direct penetration of the mercury into the blood-stream. (4) The whole dose administered is utilised. (5) The dosage is more exact. Before beginning treatment, it should be ascertained that the kidneys and teeth are healthy. The dose should be injected deeply into the muscles, and then there is little or no suffering. When intramuscular injection is not well borne (wasted subjects), and it is necessary to attack the syphilis speedily, intravenous injections may be given with advantage, and for this purpose ·01 grammes of the cyanide of mercury may be used as a dose. Glass syringes with platino-iridium needles should be used; for intravenous and intracellular injection the needles should be short and fine; for intramuscular injection the needle should be long, strong, and of fairly large diameter. To give an intramuscular injection a fold of the buttock is taken up, the needle thrust deeply in, and the solution slowly injected. If the patient complains of pain the needle must be withdrawn a little, or thrust further in. If painless at the time there should be no discomfort later. Afterwards the part must be gently massaged and any oozing swabbed away. It is best to warm the injection before giving it, as this renders it less irritating, and reincorporates any of the drug which may have been deposited on the side of the vessel. The preparations which are used may be divided into soluble and insoluble. The former admit of accurate dosage and rapid absorption, but must be repeated daily, or on alternate days. The insoluble preparations form deposits which are gradually absorbed and hence need not be repeated more often than once in five, six, or seven days. They have the disadvantages that it is not known how much is absorbed daily, and if the patient prove intolerant to the drug it is impossible to withdraw it. Liever[2] says when administered in this form it has been known to lie dormant for months and then to give rise to toxic symptoms.

For administration of the metal in the soluble form Wickham recommends the following formulæ:—

| | |
|---|---|
| Biniodide of mercury ... ... ... | ·10 grammes |
| Potassium iodide ... ... | q.s. (to dissolve the salt) |
| Sterilised distilled water... ... ... | 10 c.c. |

The initial dose should be 1 c.c. daily, to be increased gradually, keeping a careful watch all the time, until the patient is taking 3 centigrammes of the biniodide of mercury. Each course should extend over about a month. Another preparation is the solution of cyanide of mercury.

| | |
|---|---|
| Cyanide of mercury ... ... ... | ·10 grammes |
| Sterilised distilled water ... ... | 10 c.c. |

The cyanide is obtained by decomposing Prussian blue with red oxide of mercury. This is a strong preparation and 1 c.c. per diem is a sufficient dose.

The insoluble mercury may be used in the following forms:—

| | |
|---|---|
| Purified mercury ... ... | 20 grammes |
| Sterilised lanoline ... ... | 12 grammes |
| Sterilised fluid vaseline ... | q.s. to make 100 c.c. |

This should be put up in glass bulbs holding a little more than ·5 c.c. The syringes are graduated into ½ c.c., which are further divided into 10 parts. Six or seven of these should be injected for a dose.

Another preparation:—

| | |
|---|---|
| Calomel ... ... ... ... ... | 1 gramme |
| Sterilised fluid vaseline ... ... ... | 20 c.c. |

The dose of calomel for injection is ·05 to ·10 of a gramme every six or seven days. This preparation should be used in cases of gravity, when it is desirable to act vigorously. Wickham further points out the danger there is of tolerance of the drug. He advises that five months of the first year should be devoted to treatment and seven months to rest. In the second year four months should be devoted to treatment, in the third three months. Each course of treatment should extend over three or four weeks.

Karl Touton [3] advocates the treatment of syphilis at watering-places, because patients leave their homes and enter institutions and so devote their lives to their cure, social and business duties are not allowed to obtrude themselves, and fear of the nature of their disease becoming known is thrown aside. Both he and Lieven [4] advocate inunction as the best mode of presenting mercury. The former advises 33½ per cent. of mercury in a basis of resorbin, 3 to 5 grammes to be rubbed in for 10 minutes, the ointment left on the surface and not to be washed off. The latter advises unguentum cinereum (33 per cent.) to be rubbed in for 20 minutes with the same precautions. Touton shows that the waters at Wiesbaden contains a large quantity of sodium chloride, and this, on the showing of Stern,[5] Müller,[6] Voit,[7] Pauloff,[8] and Griesinger,[9] helps the therapeutic action of mercury, as also do the hydropathic measures. For the treatment at Aix-la-Chapelle Lieven [10] claims that (1) the alkalinity of the waters promotes desquamation of the effete surface layers of the epithelium, and so opens up the mouths of glands and follicles, whilst the heat determines the blood to the part, and absorption and assimilation of the mercury are facilitated; (2) the temperature at which the baths are given increases or diminishes the evaporation. The waters are taken internally, and (3) their salts ensure daily evacuation of the bowels, (4) whilst the hydrogen sulphide acts on the mercury and produces a comparatively innocuous sulphide, and so gastro-intestinal irritation is reduced to a minimum. Should mercurial symptoms show themselves, these are overcome by increasing the excretion by thermal baths, and, if necessary, withdrawing the drug for a short time. If the patient has marked nephritis, Lieven says the mercury should be first presented in the form of a pill and its effect watched. If there is an increase in the amount of albumen the mercury can be withdrawn at once, but if the albumen diminishes the drug may be pushed by inunction. Treatment should be carried out over three years, each course lasting a month or six weeks.

The first three courses should be at intervals of six months, and then if no symptoms show themselves the fourth course may be gone through at the end of the second year, and the last course at the end of the third. As a rule Lieven withholds iodides until the second year, but should symptoms show themselves they may be treated by the administration of potassium iodide by the mouth, and when absorption of the gummata begins iodipin (25 per cent. of iodine in sesomoid oil) is injected in 15-minim doses up to 10 administrations. The drug is slow in its action, but is tardily excreted, and six months after administration traces are found in the urine. Both Touton and Lieven hold that mercurial treatment should not be begun until symptoms have established themselves beyond a doubt, as a patient to avoid late manifestations must continue treatment for several years, but by taking mercury constitutional symptoms are suppressed and it may then be difficult to persuade the patient to undergo the full course. Lieven says a soft sore may be mistaken for a Hunterian chancre, and there is doubt until a roseola or mucous plaques develop.

Sir Alfred Cooper [11] advocates the Zittman treatment in cases of malignant syphilis where iodides ond mercury fail. "The principle consists in eliminating the poison from the system by sweating and purgation." The evening before the treatment is commenced the patient has a strong pill of calomel with colocynth and hyoscyamus. He is kept in bed in a room with a temperature of 80°, and his diet is a plain, ordinary one, without sugar, fruit, or spices, and throughout the day he drinks large quantities of aperient decoctions. On the fifth day he is allowed to get up, and the treatment is discontinued until the evening, when another pill is administered, and he begins another four days—and so on for 15 days.

[1] Practitioner, July 1904. [2] Ibid. [3] Ibid. [4] Ibid. [5] Berliner Klin. Woch., 1870, No. 35, and 1878, No. 5. [6] Ibid., 1870, No. 35, and Arch. d. Pharm. cxciv. [7] Physiolog. Chem. Untersuch. über die Aufnahme des Quecksilbers und seiner Verbindungen in dem Körper, 1857. p. 62. [8] Monatshefte f. prakt. Dermatol. 1892, Bd. 12, p. 332. [9] Arch. f. physiol. Heilkunde, January 6, s. 381. [10] Practitioner, July 1904. [11] Ibid.

